# Modeling dependent group judgments: A computational model of sequential collaboration

**DOI:** 10.3758/s13423-024-02619-9

**Published:** 2025-01-06

**Authors:** Maren Mayer, Daniel W. Heck

**Affiliations:** 1 Leibniz-Institut für Wissensmedien (Knowledge Media Research Center), Tübingen, Germany; 2 Department of Psychology, University of Marburg, Marburg, Germany

**Keywords:** Wisdom of crowds, Mathematical model, Advice taking, Anchoring

## Abstract

Sequential collaboration describes the incremental process of contributing to online collaborative projects such as Wikipedia and OpenStreetMap. After a first contributor creates an initial entry, subsequent contributors create a sequential chain by deciding whether to adjust or maintain the latest entry which is updated if they decide to make changes. Sequential collaboration has recently been examined as a method for eliciting numerical group judgments. It was shown that in a sequential chain, changes become less frequent and smaller, while judgments become more accurate. Judgments at the end of a sequential chain are similarly accurate and in some cases even more accurate than aggregated independent judgments (wisdom of crowds). This is at least partly due to sequential collaboration allowing contributors to contribute according to their expertise by selectively adjusting judgments. However, there is no formal theory of sequential collaboration. We developed a computational model that formalizes the cognitive processes underlying sequential collaboration. It allows modeling both sequential collaboration and independent judgments, which are used as a benchmark for the performance of sequential collaboration. The model is based on internal distributions of plausible judgments that contributors use to evaluate the plausibility of presented judgments and to provide new judgments. It incorporates individuals’ expertise and tendency to adjust presented judgments as well as item difficulty and the effects of the presented judgment on subsequent judgment formation. The model is consistent with previous empirical findings on change probability, change magnitude, and judgment accuracy incorporating expertise as a driving factor of these effects. Moreover, new predictions for long sequential chains were confirmed by an empirical study. Above and beyond sequential collaboration the model establishes an initial theoretical framework for further research on dependent judgments.

Over the last two decades, many online collaborative projects such as Wikipedia and OpenStreetMap have emerged which aim at collecting information in the form of an online encyclopedia or a comprehensive world map, respectively. Such collaborative projects use a sequential process for information collection. Essentially, one contributor starts by creating an entry, for instance, a Wikipedia article or an OpenStreetMap object, which can then be adjusted or maintained by subsequent contributors encountering it. This incremental process of sharing and aggregating information yields highly accurate results, both for factual information in Wikipedia (Giles, 2005) and for geographical representations in OpenStreetMap (Girres & Touya, 2010).

Recently, Mayer and Heck (2024) examined this process termed sequential collaboration systematically as a method for judgment aggregation (see also Miller & Steyvers, 2011). In these studies, participants were assigned to sequential chains of four or six contributors. One after another, they were consecutively presented with numerical judgments of the previous participants for general knowledge questions or city locations. Each contributor only saw the most recent judgment but did neither know their position in the sequential chain nor the complete history of all judgments. Contributors’ task was to decide whether to adjust or maintain the presented judgment in the sequential chain which was then either replaced with their revised judgment or maintained.

Mayer and Heck (2024) found that, over the course of a sequential chain, both the probability that contributors adjust a presented judgment and the average magnitude of such adjustments decrease. Most importantly, on average across multiple groups, the accuracy of judgments increased over the course of a sequential chain. As a benchmark, the accuracy of the final judgments at the last position in the sequential chain can be compared against the unweighted average of an identical number of independent judgments (wisdom of crowds, Surowiecki, 2004). Results showed that sequential collaboration yields similarly accurate and in some cases even more accurate estimates than unweighted averaging. This is an important and interesting finding as the unweighted average of independent judgments already yields highly accurate estimates in various contexts and tasks (Hueffer et al., 2013; Larrick & Soll, 2006; Steyvers et al., 2009).

A subsequent study examined a possible mechanism for the successful aggregation of dependent judgments, showing that sequential collaboration results in an implicit weighting of judgments by participants’ expertise (Mayer et al., 2023). In three experiments, contributors with higher expertise adjusted the presented judgments more frequently and provided larger improvements than contributors with lower expertise. Moreover, larger deviations between the presented judgment and the correct answer led to more frequent adjustments to the presented judgment and larger improvements. Finally, the more and the later contributors with high expertise entered a sequential chain, the more accurate the final estimates at the end of sequential chains were.

Recently, Rebholz et al. (2024) reanalyzed data from Mayer and Heck (2024). Using a model-based approach, they found that the presented judgments are strongly integrated into individuals’ judgments. The authors hypothesized that this is due to the possibility to opt-out of providing a judgment in sequential collaboration. The result that individuals do not ignore presented judgments stands in contrast to findings of advice taking literature which typically showed egocentric discounting of advice (Bonaccio & Dalal, 2006). Moreover, Rebholz et al. (2024) did not find any differences in the extent of advice taking over the course of a sequential chain even though the variability in the extent of integration of the presented judgment decreases over the course of a sequential chain.

The studies outlined above provide first insights into sequential collaboration as a process of judgment aggregation and show that the possibility to opt-out of giving a judgment may be important for the continuous improvement of dependent judgments. Even though these initial results and conclusions are promising, a formal theory of sequential collaboration is currently lacking. Computational models formalize assumptions and findings about psychological processes. While being precise, they also capture reality only partially, are typically incomplete, and simplify existing relationships and mechanisms. Nonetheless, models provide an important tool for formalizing assumptions, clarifying necessary boundary conditions, exposing contradictory ideas, and deriving new, testable predictions (Smaldino, 2017; Villarreal et al., 2023).

To advance theory building, the present work proposes a computational model of sequential collaboration. Thereby, we formalize the assumptions originally made by Mayer and Heck (2024) for deriving hypotheses about the performance and the underlying mechanisms of sequential collaboration. The model builds on verbal theories from related research on wisdom of crowds (Surowiecki, 2004), anchoring (Tversky & Kahneman, 1974), and advice taking (Bonaccio & Dalal, 2006). In sequential collaboration, we are interested in judgment formation and individuals’ decision-making of whether and how presented judgments are adjusted. The model contributes to a better understanding of the phenomenon of sequential collaboration as a mechanism of judgment aggregation and embeds it into existing theories and findings. The model also applies to independent individual judgments, which provide a benchmark for the accuracy of sequential collaboration. Modeling both types of judgments within the same formal framework allows us to model and compare predictions across various settings.

**Fig. 1:**
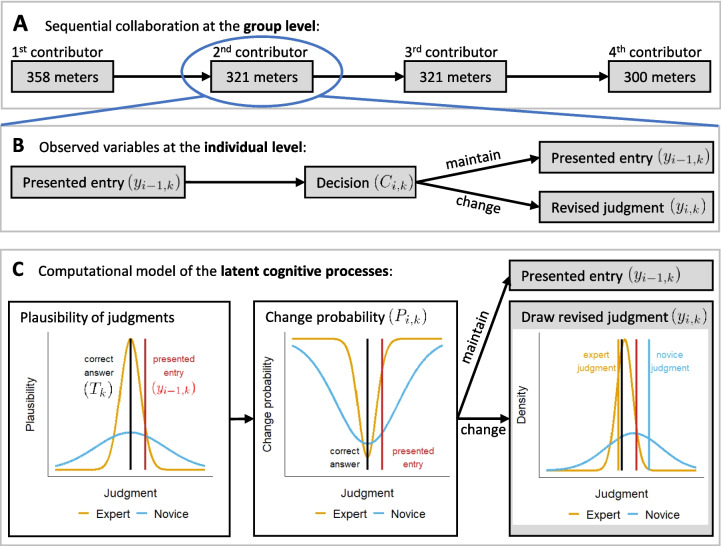
Model of sequential collaboration

In the following, we first develop a computational model that applies both to independent judgments in standard wisdom-of-crowds paradigms and dependent judgments in sequential collaboration. The computational model serves an explanatory purpose by describing known phenomena, formalizing theoretical assumptions, and deriving novel predictions, but it is not a statistical model that is fitted to data. To provide a first check of the validity of the model, we performed a simulation study showing that the model is in line with empirical findings of previous studies. In a second simulation study, we modified the model parameters to derive novel predictions for the performance of sequential collaboration in long sequential chains (i.e., groups of 20 or more instead of only 4 or 6 individuals). We also report an empirical study testing the novel model predictions for change probability, change magnitude, and judgment accuracy. Finally, we discuss the implications and possible extensions of the model.

## A computational model of sequential collaboration and independent judgments

The proposed computational model of sequential collaboration operates at two levels. At the individual level, the model describes the cognitive processes of judgment formation within a single sequential step in the collaborative process. Given the judgment of a previous contributor, the model predicts the decision of whether the presented judgment is maintained or adjusted, and in the latter case, how the judgment is revised. The model considers differences in item difficulty. Moreover, it assumes that contributors differ in expertise and have a certain response bias to generally adjust presented judgments. Thereby, the model integrates aspects and ideas from cultural consensus theory (Anders et al., 2014; Mayer & Heck, 2023), dialectical bootstrapping (Herzog & Hertwig, 2009, 2014), and item response theory (Embretson & Reise, 2000).

At the group level, the predictions for judgments at the individual level are used to simulate a sequential chain of sequential steps in which each contributor encounters the judgment of the previous one. The model also makes predictions about independent judgments, which are required to account for the initial judgments of the first contributor in a sequential chain (who does not see any previous judgment). Modeling both independent and dependent judgments within a single, unifying framework allows us to compare the predicted accuracy of sequential collaboration to that of aggregated independent judgments (i.e., the standard paradigm in wisdom of crowds, Surowiecki, 2004) on an equal footing.

Figure 1 displays the model for judgments in sequential collaboration. Figure 1A shows an exemplary sequential chain of judgments for the general knowledge question “How tall is the Eiffel tower?” at the group level. Figure 1B shows the observed variables in one sequential step at the individual level. Finally, Fig. 1C illustrates the assumed cognitive processes underlying sequential collaboration. In the following, we first specify a simple model for independent judgments before extending this model to dependent judgments in sequential collaboration.

### Modeling independent judgments

In line with common assumptions about the wisdom of crowds (Davis-Stober et al., 2014; Larrick & Soll, 2006), the model assumes that individuals have a subjective, internal distribution of plausible values for each question representing intra-individual variability in judgments (Herzog & Hertwig, 2009, 2014). Moreover, we build on item response theory (Embretson & Reise, 2000) and cultural consensus theory (CCT, Anders et al., 2014; Mayer & Heck, 2023) in assuming that a combination of item difficulty and person’s ability determines the accuracy of a judgment. The resulting baseline distribution of plausible values for an item $k$ is assumed to follow a normal distribution. If an individual $i$ is asked to provide an independent judgment for item $k$, the judgment $y_{ik}$ is generated based on the distribution of plausible values,

1 $$\begin{aligned} Y_{ik} \sim \text {Normal}(\mu = T_k, \sigma = D_k E_i). \end{aligned}$$

In the following, we explain the meaning of the three parameters.

The mean of the baseline distribution of plausible values is determined by the correct numerical answer $T_k$ for item $k$. This assumption is in line with findings on wisdom of crowds showing that individuals produce unbiased judgments on average (Larrick & Soll, 2006; Steyvers et al., 2009). Whereas there is ample evidence for the validity of wisdom of crowds in general (Davis-Stober et al., 2014; Larrick & Soll, 2006; Surowiecki, 2004), this assumption may be inadequate under certain conditions. For instance, judgment processes can lead to non-normal distributions (Hueffer et al., 2013; Lorenz et al., 2011; Lorenz, 2021) or extreme judgments (Thomas et al., 2021) in which the mean of the judgments does not longer reflect the correct answer. However, for the present purpose of comparing the accuracy of independent and dependent judgments, this is not a critical assumption given that it is made both for wisdom of crowds and for sequential collaboration (skewed distributions are considered in the empirical study below).

The standard deviation of the baseline distribution of plausible values is determined by the product of the difficulty $D_k$ of item $k$ and the expertise $E_i$ of contributor $i$. Since the standard deviation must be positive, both $D_k$ and $E_i$ are restricted to be positive. In test theory, item difficulty is related to the probability that respondents provide the correct answer to a question (Embretson & Reise, 2000; Lord et al., 1968). Here, we adopt this psychometric concept for numerical judgments, assuming that the width of the baseline distribution of plausible values determines the accuracy of independent judgments (Anders et al., 2014). Essentially, larger (smaller) values of $D_k$ indicate a more difficult (easier) item since the discrepancies of individual judgments from the correct answer increase (decrease) as the standard deviation becomes larger (smaller).

Expertise is a domain-specific concept (Herling, 2000) which describes abilities, knowledge, or skills of an individual (Herling, 2000; Schulze & Krumm, 2017) that lead to improved performance in domain-related tasks (Budescu & Chen, 2014; Mayer & Heck, 2023; Merkle et al., 2020). Similar to item response and cultural consensus theory, contributors’ expertise $E_i$ reflects differences in knowledge and skills between individuals. Lower values of $E_i$ lead to a smaller standard deviation and thus more accurate judgments closer to the correct answer $T_k$. In contrast, larger values of $E_i$ lead to an increased standard deviation, and thus, to less accurate judgments with a larger dispersion around the correct answer $T_k$. It is necessary that the values for expertise are always positive to obtain a proper standard deviation for the distribution of plausible values. As a remedy, the unbounded expertise values $E^*_i$ are transformed using the (inverse) logistic link function similar as in logistic regression and item response theory,

2 $$\begin{aligned} E_{i} = \frac{1}{1 + e^{- E^{*}_{i} }}. \end{aligned}$$

The (inverse) logistic transformation ensures that the values of $E_i$ are always positive and range between 0 (high expertise) and 1 (low expertise). The restriction to the unit interval $[0,1]$ is an important feature for modeling sequential collaboration in the next section.^1^

We assume that latent expertise follows a normal distribution on the real line,

3 $$\begin{aligned} E^*_{i} \sim \text {Normal}(\mu = 0, \sigma = \sigma _{E^*_i}). \end{aligned}$$

By centering the distribution of latent expertise, item difficulty $D_k$ can be interpreted as the standard deviation of the plausible-value distribution for a contributor with average expertise (i.e., if $E_i = 1/(1 + e^{0}) = 1$).

### Modeling dependent judgments in sequential collaboration

As shown in Fig. 1B, the model assumes that contributors go through a two-stage process. When contributors encounter the judgment of a previous contributor, they first decide whether to adjust or maintain the presented judgment. We assume that this decision is based on how plausible a contributor considers the presented judgment to be. Only if the contributor decides to adjust the presented judgment they provide a revised judgment. Otherwise, the presented judgment by the previous contributor is maintained and simply passed on to the next contributor in the sequential chain.

Figure 1C illustrates the most important assumptions regarding the cognitive processes underlying sequential collaboration. The left plot of Fig. 1C shows the baseline distribution of plausible values for two contributors: a peaked distribution resembling high expertise (orange color) and a wide distribution resembling low expertise (blue color). Moreover, the two vertical lines illustrate the correct answer to the item (black color) and the presented judgment of the previous contributor (red color). The second plot of Fig. 1C depicts the probability of adjusting the presented judgment, denoted as change probability in the following. The more the presented judgment is in line with the baseline distribution of plausible values, the more likely a contributor will maintain it with a certain probability (i.e., the change probability will be relatively small). If the distance of a presented judgment to the center of the plausibility distribution is large, it is considered to be implausible and likely to be adjusted (i.e., the change probability will be larger). Finally, when a contributor decides to adjust the presented judgment, a new, revised judgment is drawn from an updated distribution of plausible values which is shifted towards the presented judgment (see right plot of Fig. 1C).

The model assumes that contributors evaluate a presented judgment based on the baseline distribution of plausible judgments (Eq. 1). To be precise, they assess how likely the presented judgment of a previous contributor $y_{i-1,k}$ is in light of the distribution of plausible values. Presented judgments are considered to be more plausible the closer they are to the mean of the distribution; conversely, they are considered to be less plausible the further away they are from the mean. This evaluation process is formally modeled by transforming the cumulative distribution function of the baseline plausible-value distribution in a suitable way (Fig. 1C). More precisely, the probability $P_{ik}$ that a contributor $i$ adjusts the presented judgment $y_{i-1,k}$ of the previous contributor $i-1$ for item $k$ is defined as

4 $$\begin{aligned} P_{ik} \sim G_i + (1-G_i) \,4 \, \left[ \Phi \left( \frac{y_{i-1,k} - T_k}{D_k E_i}\right) - \frac{1}{2} \right] ^2. \end{aligned}$$

At the core of Eq. 4 is the cumulative distribution function $\Phi$ of the standard normal distribution. The distribution function of plausible values is scaled to have mean $T_k$ and standard deviation $D_k E_i$ (cf. Equation 1). The cumulative probability ranges from 0 to 1 with an inflection point at the mean of the distribution, where the probability is exactly 1/2. As illustrated in the center plot of Fig. 1C, the model assumes that the predicted change probability has a minimum at the mean $T_k$ of the baseline distribution of plausible values. Moreover, change probabilities range from zero for plausible presented judgments, which are unlikely to be adjusted to one for implausible presented judgments, which are likely to be adjusted. To ensure these properties, we apply a quadratic transformation to the cumulative distribution function. By subtracting 1/2 from the cumulative probability and squaring the result, we obtain a function with a value of zero at the mean $T_k$ and monotonically increasing values the further one moves away from the mean. Since the quadratic transformation returns values between 0 and 1/4, we multiply the result by four to obtain values in the interval $[0,1]$.

The model assumes that each contributor has a certain tendency to generally adjust presented judgments irrespective of their expertise or the item content. This general tendency to change presented judgments reflects conservative and liberal response biases for the decision to adjust or maintain a presented judgment. The idea of a general response bias is a common assumption in many modeling approaches such as signal detection theory which aims at disentangling an individual’s detection ability from their response criterion (Lynn & Barrett, 2014; Pastore & Scheirer, 1974). Similarly, in judgment and decision making, individuals differ in the extent of accepting advice (Bonaccio & Dalal, 2006; Rebholz et al., 2024) and the extent of showing an anchoring bias (Cheek & Norem, 2022; Tversky & Kahneman, 1974).

In the present model, response tendencies affect the change probability in Eq. 4 via the parameter $G_i$ which is the baseline probability of a contributor $i$ adjusting presented judgments. The probability $G_i$ is person-specific but does not depend on any item properties. Similar to individuals’ expertise, the tendency to change presented judgments is first transformed using the (inverse) logistic link function such that the values of $G_i$ range between zero and one,

5 $$\begin{aligned} G_{i} = \frac{1}{1 + e^{-G^*_i}}. \end{aligned}$$

Similarly to expertise, the distribution of latent response tendencies is modeled with a normal distribution. We expect that individuals generally have a change tendency below 50%, and thus, we assume a mean of $-1$ for the normal distribution,

6 $$\begin{aligned} G^*_{i} \sim \text {Normal}\left( \mu = -1, \sigma = \sigma _{ G^*_i}\right) . \end{aligned}$$

The decision $C_{ik}$ whether to adjust or maintain the presented judgment $y_{i-1,k}$ of the previous contributor is sampled from a binomial distribution based on the change probability $P_{ik}$. Whereas $C_{ik} = 0$ denotes the decision to maintain the presented judgment, $C_{ik} = 1$ denotes the decision to revise the judgment,

7 $$\begin{aligned} C_{ik} \sim \text {Binomial}\left( p = P_{ik} \right) . \end{aligned}$$

Given the decision $C_{ik}=0$, the presented judgment $y_{i-1,k}$ is maintained and the process at the individual level is completed. Given the decision $C_{ik}=1$, the contributor provides a revised judgment $y_{i,k}$ from an updated distribution of plausible values. Importantly, the updated distribution of plausible values differs from the baseline distribution of evaluating the plausibility of a presented judgment due to the model’s assumption that the process of evaluating the presented judgment affects the assessment of which values are considered to be plausible.

Compared to the baseline distribution of plausible values, the updated distribution of plausible values differs in two ways. First, we assume that evaluating the judgment of a previous contributor leads to anchoring, the robust phenomenon that the presentation of unrelated numerical values influences subsequent numerical judgments (Röseler & Schütz, 2022; Schley, 2023; Tversky & Kahneman, 1974). For instance, letting participants determine a high or low random number from a wheel of fortune influences their subsequent judgment of the proportion of African countries in the United Nations (Tversky & Kahneman, 1974). In sequential collaboration, the presented judgment of a previous contributor is clearly relevant for the task, and thus, evaluating the given value is likely to induce anchoring. Specifically, our model assumes that presented judgments affect contributors’ perception of what are plausible answers. Accordingly, revised judgments, which are generated based on the updated distribution of plausible judgments, will be shifted towards the presented judgment of the previous contributor. Such an anchoring effect is likely smaller for contributors with higher expertise. In contrast, contributors with lower expertise have less knowledge and may thus be drawn more strongly towards the presented anchor (Mayer & Rebholz, 2024; Smith et al., 2013; Smith & Windschitl, 2015; Wilson et al., 1996). The assumption that the degree of anchoring in sequential collaboration depends on individuals’ expertise is supported by previous studies in which contributors’ expertise was both measured and manipulated (Mayer et al., 2023).

Second, by showing a judgment of a previous contributor, sequential collaboration offers the potential advantage of providing a frame of reference for making revised judgments. In general, providing contextual information in a judgment task is known to have beneficial effects for subsequent judgments (Brown & Siegler, 1996). Research on seeding effects demonstrates that providing individuals with information closely related to the judgment task results in more accurate judgments (Groß et al., 2023). For instance, providing individuals with the size of the population of Cameroon improves their accuracy of judging the size of the population of Peru. A similar effect was observed for groups working on numerical-judgment tasks (Laughlin et al., 1999). Group judgments become more accurate when groups are provided with a numerical value of a closely related quantity which serves as a frame of reference. For instance, providing the length of the Nile as a frame of reference when asking for the length of the Mississippi led to fewer extreme judgments compared to the absence of any frame of reference (Bonner et al., 2007). We assume that the same mechanism also improves the accuracy of revised judgments in sequential collaboration compared to independent judgments. Essentially, a contributor may use the presented judgment of a previous contributor as a frame of reference. The model assumes that the standard deviation of the updated distribution of plausible values in sequential collaboration is smaller than that of the baseline distribution, implying that extreme judgments become less likely.

The updated distribution of plausible values $Y^*_{ik}$ in Eq. (8) is similar to the baseline distribution of plausible values in Eq. (1). However, the former takes into account that the initial evaluation of the presented judgment results an updated distribution potentially leading to more accurate judgments due to anchoring and having a frame of reference,

8 $$\begin{aligned} Y^*_{ik} \sim \text {Normal}\left( \mu = T_k + A_{ik}, \sigma = R D_k E_i \right) . \end{aligned}$$

The mean of the resulting updated distribution in sequential collaboration is determined by two components: the correct answer $T_k$ for item $k$ and the anchoring bias $A_{ik}$. The anchoring value $A_{ik}$ induces an additive shift towards the presented judgment $y_{i-1,k}$, while the amount of anchoring is scaled by the expertise $E_i$ of the contributor,

9 $$\begin{aligned} A_{ik} = E_i \left( y_{i-1,k} - T_k \right) . \end{aligned}$$

To serve as a scaling factor, it is important that contributors’ expertise $E_i$ can only take on values between 0 (high expertise) and 1 (low expertise), as ensured by the logistic link function in Eq. 2. The model predicts that contributors with high expertise ($E_i \approx 0$) are only slightly affected by the presented judgment whereas contributors with low expertise ($E_i \approx 1$) are strongly affected by anchoring.

The distribution of independent judgments (Eq. 1), which is also used to evaluate the plausibility of presented judgments, only depends on expertise and item difficulty. In contrast, the standard deviation of the distribution of revised judgments in Eq. 8 is extended by the multiplicative scaling factor $R < 1$ which reflects the effect of the presented judgment providing a frame of reference. The smaller the value of $R$, the larger the presented judgment affects the revised judgment.

The model described above outlines the hypothesized cognitive processes underlying one step in sequential collaboration. Based on this process at the individual level, chains of judgments can be modeled sequentially by using the outcome of one sequential step as input for the next one. The model thereby mimics the process of judgment formation in studies and applications on sequential collaboration. Furthermore, our computational model makes predictions about independent individual judgments, which are required as starting values for sequential chains of dependent judgments (Mayer & Heck, 2024). Thereby, it facilitates a comparison of sequential collaboration with the aggregation of independent judgments. Specifically, computing an unweighted average of independent individual judgments (often referred to as wisdom of crowds in a narrow sense) can serve as a benchmark for evaluating the predicted performance of sequential collaboration in different contexts.

## Accounting for established phenomena in sequential collaboration

In the following, we show that the proposed computational model is in line with empirical findings for sequential collaboration. We first focus on characteristic patterns of judgments in sequential chains and on the role of expertise in sequential collaboration. In a second step, we derive new predictions for the development of judgment accuracy in long sequential chains, while using the unweighted averaging of independent judgments in larger crowds as a baseline for comparison.

In our first simulation study, we assess whether the proposed model can account for empirical results on change probability, change magnitude, and accuracy of judgments in sequential collaboration and unweighted averaging of independent judgments (Mayer & Heck, 2024). Moreover, we show that, according to the model, expertise is a key determinant of whether and how judgments of previous contributors are revised which has implications for the optimal composition of sequential chains (Mayer et al., 2023).

We used the proposed model to simulate chains of dependent judgments in sequential collaboration. We set the standard deviation of expertise to $\sigma ^*_{E} = 1$ and the standard deviation of contributors’ change tendency to $\sigma ^*_{G} = 1$, assumed a correct answer $T_k = 0$ for all items and a reduction of the standard deviation (i.e., a frame-of-reference effect) of $R = 0.4$. Moreover, we varied item difficulty $D_k$ between 1 and 5. These parameters were selected to reflect previous empirical studies as closely as possible.

Based on these parameters, we simulated judgments for an experiment with 60 items and 100 groups of individuals (i.e., sequential chains) with four individuals per group (i.e., the chain length) resulting in 400 participants and 24,000 individual trials. Regarding the person parameters, we used normal distributions to sample expertise values with the standard deviation $\sigma ^*_{E}$ (see Eq. 3) and response-bias parameters (which reflect the tendency to change presented judgments) with the standard deviation $\sigma ^*_{G}$ from Eq. 6. To simulate items with an empirically plausible distribution, the item difficulty $D_k$ was sampled from a uniform distribution, $D_k \sim \text {Uniform}(1,5)$. With these settings, we generated sequential chains of judgments according to the model outlined above. Only one simulation was conducted to compare model predictions against the results of previous experiments on sequential collaboration. Hence, all parameters were identical for all empirical studies the model is compared to. To test the robustness of the model, the effect of specifying different parameter values for $\sigma ^*_{E}$, $\sigma ^*_{G}$, and $R$ are presented in Appendix A.

**Fig. 2:**
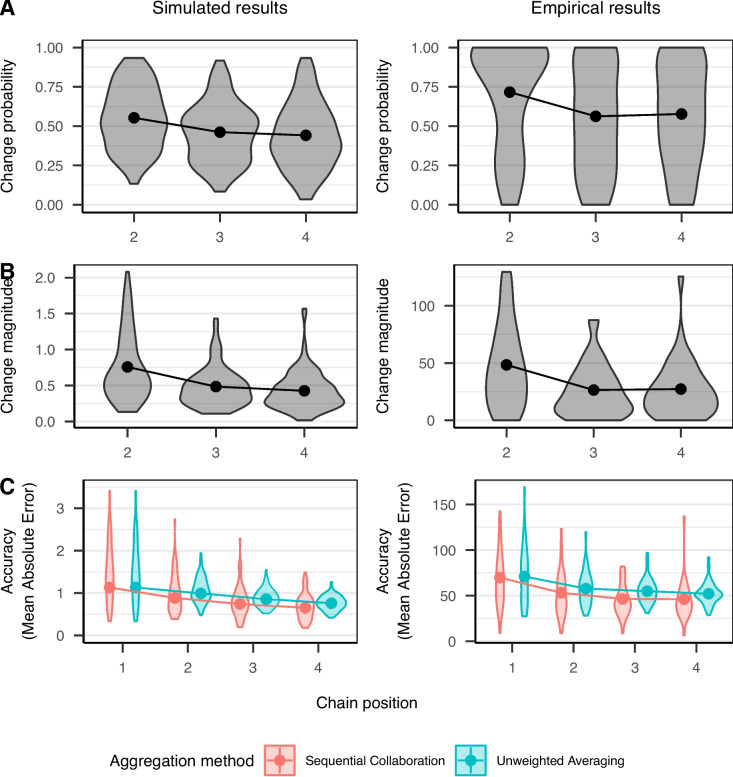
Results for simulated data from the computational model and empirical data of Experiment 3 from Mayer and Heck (2024). *Note.* *Points* display empirical means with the corresponding 99% between-subjects confidence intervals mostly being not visible. *Violin plots* indicate the distribution of the dependent variable aggregated across items within each contributor. The plot for change magnitude only includes presented judgments that were adjusted by contributors

### Predicted adjustment decisions and judgment accuracy

Figure 2 displays the results of the simulation and empirical data of Experiment 3 of Mayer and Heck (2024) with the dependent variables change probability (A), change magnitude (B), and judgment accuracy (C). We only present the results of Experiment 3 because the first two experiments by Mayer and Heck (2024) provided qualitatively similar patterns of results.

In the simulation study, change magnitude was computed as the absolute difference between the presented judgment and the simulated judgment (if a revised judgment was made at all). Judgment accuracy was computed as the absolute value of each judgment because the correct answer for each simulated item was zero ($T_k = 0$).

In the empirical study, participants were randomly assigned to two conditions in which they provided either independent judgments or dependent judgments after being presented with a judgment of another participant. Some of the independent judgments served as starting values for the sequential chains. The remaining independent judgments were aggregated by computing the mean, a commonly used benchmark for judgment accuracy in the literature on wisdom of crowds (Larrick & Soll, 2006). The task was to position 57 cities on seven different European maps which had a size of $800\times 500$ pixels. Accordingly, change magnitude and judgment accuracy were computed as the Euclidean distance (measured in pixels) between the presented judgment and (a) the revised judgment or (b) the correct answer, respectively.

The computational model of sequential collaboration predicts that change probability decreases with increasing chain position. This is due to the fact that contributors encounter increasingly accurate judgments along the sequential chain which are less likely to be adjusted by all contributors irrespective of their expertise. As shown in Fig. 2A, a decrease in change probability over the course of a sequential chain was indeed observed in the empirical study by Mayer and Heck (2024).

Model predictions and empirical data also matched for change magnitude. The model predicts a continuous decrease in change magnitude over the course of a sequential chain. Since judgment accuracy is predicted to increase, adjustments to the presented judgments do not need to be as large as for the early, less accurate judgments in the sequential chain. The results of Experiment 3 by Mayer and Heck (2024) in Fig. 2B were in line with these model predictions, showing a decreasing change magnitude over the course of a sequential chain.

Regarding accuracy, the model predicts an increasing performance of judgments, that is, a decreasing mean absolute error of dependent judgments (see Fig. 2C). The predicted accuracy of sequential collaboration is even higher than that of the unweighted averaging of independent judgments. Judgment accuracy in sequential collaboration is assumed to be fostered by the possibility to opt-out of providing (possibly ill-informed and misleading) judgments. Only if contributors are sufficiently confident, they provide judgments according to their expertise. Figure 2C also shows that the distribution of judgments in sequential collaboration has slightly more variation at the final chain position than unweighted averaging, probably due to more frequent outliers. Nonetheless, the judgments obtained with sequential collaboration are still closer to the correct answer (i.e., the assumed truth with a value of zero). The empirical data in Fig. 2C closely resemble these patterns. In Experiment 3 by Mayer and Heck (2024), the accuracy of judgments in sequential collaboration was higher than that of the unweighted averaging of independent judgments. Moreover, the empirical distribution of dependent judgments in sequential collaboration showed a relatively small variance. Importantly, both the empirical and the simulated data showed that, compared to the aggregation of an equal number of independent judgments, sequential collaboration yielded a higher judgment accuracy especially in the first steps of the sequential chain. The longer the sequential chains (i.e., the larger the group), the smaller the difference in judgment accuracy between the two forms of judgment aggregation became.

**Fig. 3:**
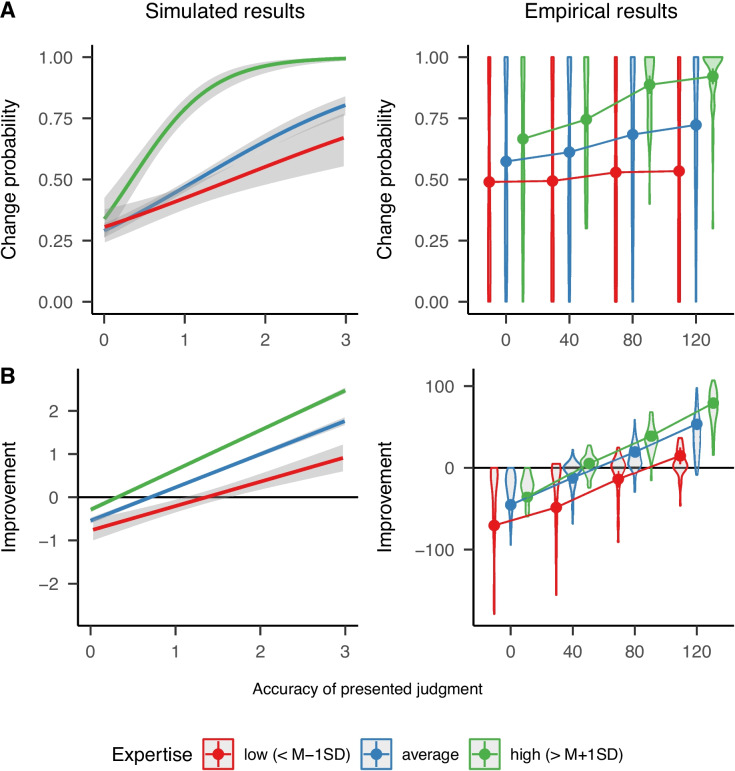
Results for simulated data from the computational model and empirical data of Experiments 1 and 3 from Mayer, Broß, & Heck (2023). *Note.* *Curves* indicate the fitted results for (generalized) linear mixed models applied to the continuous dependent variable of presented accuracy in simulated data. *Points* display empirical means with error bars showing the corresponding 99% between-subjects confidence intervals. *Violin plots* indicate the distribution of the dependent variable aggregated across items within each contributor

### The role of expertise in dependent judgments

#### Modeling a sequential step

Figure 3 compares the results of the simulation with the empirical results of Experiment 1 from Mayer et al. (2023). Simulated results reported here are obtained from the same simulation described above. The two dependent variables of change probability (A) and improvement in accuracy (B) were observed in this experiment for only one step in a sequential chain (i.e., for a single dependent judgment conditionally on seeing a previous judgment).

Experiment 1 used the same map materials as Experiment 3 of Mayer and Heck (2024). To measure participants’ expertise, we elicited independent judgments for 17 of the 57 cities. Each of the remaining 40 cities was presented with a hypothetical previous judgment that had a predetermined, randomly chosen accuracy (operationalized as the distance to the correct answer with four levels of 0, 40, 80 or 120 pixels). By manipulating the accuracy of the presented judgments, one can control nuisance variables that may occur in natural sequential chains. In the empirical study, improvement in judgment accuracy was computed as the difference between the accuracy of presented and revised judgments (if a revised judgment was provided at all). Accuracy was again computed as the Euclidean distance to the true position of a city (measured in pixels).

Concerning the model, we aimed at simulating predictions for a scenario that resembles the structure of Experiment 1 by Mayer et al. (2023) as closely as possible. For this purpose, we only included simulated participants at chain position two in the analysis. Moreover, we used the first 20 simulated independent judgments and computed the mean judgment accuracy of these judgments as a proxy for contributors’ expertise. Improvement of judgments was computed as the difference between the accuracy of the presented judgment and the accuracy of the revised judgment (if a revised judgment was provided at all). Thus, the variable “improvement” takes on positive values if the presented judgment becomes more accurate and negative values if the presented judgment is worsened.

Figure 3A shows the change probability as a function of contributors’ expertise for the simulated and the empirical data by Mayer et al. (2023). The model predicts that contributors high in expertise are more likely to adjust presented judgments compared to contributors low in expertise. As indicated by the monotonically increasing slopes, contributors are expected to be more likely to adjust presented judgments the more the judgments deviate from the correct answer. The predicted patterns show that the possibility to opt-out of a presented judgment allows contributors to contribute more or less depending on their level of expertise: On the one hand, high-expertise contributors are generally more likely to adjust presented judgments and they can contribute even to presented judgments that are already accurate. On the other hand, low-expertise contributors are less likely to adjust judgments, both because they cannot detect small inaccuracies and because they can contribute only to judgments that are quite inaccurate. These predicted patterns were also found empirically given that the change probability increased both with contributors’ expertise and the accuracy of presented judgments. Moreover, the increase in change probability for increasingly inaccurate presented judgments is slightly stronger for contributors with high than those with low expertise. The model-based simulations also showed that contributors with high expertise are predicted to change already correct judgments more frequently than contributors with low expertise. Note that Mayer et al. (2023) made exactly the opposite prediction, assuming that experts could spot and maintain correct answers more easily than novices. However, the computational model reveals exactly the pattern empirically observed.

Figure 3B displays the model predictions and empirical findings for the improvement of the revised compared to the presented judgments. The model predicts that the higher contributors expertise, the more they can improve presented judgments if they choose to make an adjustment. Conditional on making an adjustment, contributors higher in expertise are predicted to worsen presented judgments that are already highly accurate less than contributors with lower expertise. Last, the model predicts an increase in improvement the less accurate the presented judgment is. Patterns similar to the model predictions emerged in the empirical study by Mayer et al. (2023), showing that contributors higher in expertise indeed improved the presented judgments more and worsened correct judgments less than contributors with lower expertise.

**Fig. 4:**
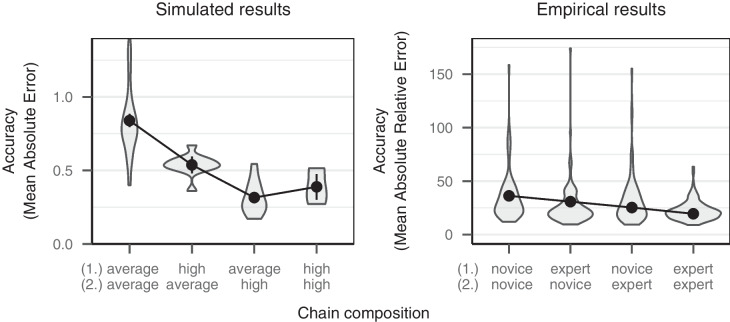
Results for simulated data from the computational model and empirical data of Experiment 3 from Mayer, Broß, & Heck (2023). *Note.* *Points* display empirical means with *error bars* showing the corresponding 99% between-subjects confidence intervals. *Violin plots* indicate the distribution of the dependent variable aggregated across items within each contributor

#### Modeling a sequential chain

The influence of expertise on the accuracy of judgments in sequential collaboration can be assessed in more detail by considering chains with different compositions of experts and novices. Experiment 3 of Mayer et al. (2023) used a random-dots estimation task (Honda et al., 2022) for which participants were either trained in a technique that made estimating dots easier and more accurate (experts) or received no training (novices). The main goal of Experiment 3 by Mayer et al. (2023) was to compare the accuracy of sequential chains with different compositions of experts and novices. More precisely, participants were assigned to sequential chains which consisted of either two novices, two experts, or one expert and one novice. Sequential chains were started by presenting preselected values, thereby only having participants at chain positions two and three. Since the correct number of presented dots varied between 100 and 599, we computed the relative accuracy of judgments to make judgments comparable. Relative accuracy was computed as the absolute difference between the presented judgment and the correct answer divided by the correct answer. To obtain percentage values, the result was multiplied by 100.

To derive model predictions for this experimental design, we selected chain positions two and three from the simulation. Again, expertise was computed based on the accuracy for independent judgments for the first 20 items. Based on this computed expertise, we selected simulated participants having either average expertise ($-1SD \le M \ge 1SD$) or high expertise ($M > 1SD$). Thus, four different chain compositions emerged resembling the design of the empirical results.

Figure 4 depicts the accuracy of the final judgment by the last contributor in a sequential chain with three participants. As shown in the left panel, the model predicts that sequential chains yield most accurate estimates the more and the later contributors with high expertise enter the sequential chain. Essentially, contributors are better at detecting incorrect judgments the higher their expertise because their internal distribution of plausible values is both narrower (i.e., more accurate) and less influenced by the presented judgment (i.e., there is less anchoring). These effects lead to an increasing probability of changing the presented judgment and contributing a revised judgment with a higher accuracy. In line with these predictions, the empirical data in the right panel showed that the more and the later contributors with high expertise entered the sequential chain, the more accurate the obtained estimates were.

Overall, the computational model predicts data patterns for sequential collaboration and the unweighted averaging of independent judgments that capture core features of previous studies. The simulated data closely resembled empirical findings of change probability, change magnitude, and judgment accuracy. Over a sequential chain, judgments are less frequently adjusted and these adjustments become smaller which ultimately leads to increasingly accurate judgments. Moreover, both simulated and empirical data showed a positive effect of expertise on judgments in sequential collaboration. With increasing expertise, contributors are more likely to adjust and improve presented judgments. This behavior results in increasingly accurate estimates, the more and the later experts enter sequential chains.

Even though the computational model showed promising results in terms of accounting for previously known phenomena, the described model has some limitations. First, the empirical studies were conducted before developing the model. Thus, predictions were not derived from the model and then tested empirically in a confirmatory fashion. Instead, the model was developed on the basis of those data later used for validation. To overcome this limitation, we derive new model predictions in the next section and test one of these predictions empirically.

Moreover, the predicted patterns rest on several free parameters drawn from parametric distributions that were adjusted to approximate the qualitative patterns observed in previous empirical studies. For instance, the person parameters $E_i$ for contributors’ expertise and the individual tendency $G_i$ to generally adjust the presented judgments were drawn from normal distributions. Moreover, the item difficulty $D_k$ was drawn from a uniform distribution with set lower and upper bounds, whereas the reduction $R$ of the standard deviation of revised judgments in sequential collaboration was a fixed value. Appendix A provides additional plots showing that our model and its predictions are robust to adjustments in these choices of parameter values.

## Novel predictions for long sequential chains

Besides replicating previous empirical findings, the proposed computational model also allows us to make novel predictions that have not yet been investigated empirically. The model predicts that sequential collaboration leads to more accurate results compared to unweighted averaging when the variation in expertise ($\sigma ^*_E$) is large. This advantage of sequential collaboration decreases the more the variation in expertise decreases. According to the assumptions of the model, this is due to the fact that sequential collaboration incorporates a mechanism for implicitly weighing judgments by expertise (Mayer & Heck, 2024). The possibility to opt-in or opt-out of providing a judgment allows contributors with high expertise to make accurate adjustments, which are likely to improve the presented judgments. In contrast, contributors with lower expertise do not necessarily have to revise the presented judgments, which would possibly lead to a decrease in accuracy. For this selection mechanism to be effective, a certain variance in expertise between contributors is required. If the variation in expertise is large, sequential collaboration helps to include judgments only of those contributors with high expertise. In contrast, if the variation in expertise between individuals is small, each decision to opt in or out of providing a judgment has a similar effect on the accuracy of the provided judgment.

In the following, we derive a novel prediction from the computational model about the performance of sequential collaboration in long chains (i.e., for large groups of contributors). In previous research, sequential collaboration has mostly been examined in relatively short chains of up to six contributors (Mayer et al., 2023; Mayer & Heck, 2024). One exception is Miller and Steyvers (2011) who showed that participants performing sequential collaboration provided more accurate answers to rank order tasks than the aggregate of participants providing independent judgment with few long chains comprising up to 65 contributors. Whereas sequential collaboration provides accurate results for small groups, it likely does not outperform unweighted averaging of independent judgments in larger groups. This can already be observed in Fig. 2C where the accuracy of unweighted averaging increases more strongly as the groups become larger compared to the increase in accuracy of sequential collaboration as the length of sequential chains increases. Due to the central limit theorem, unweighted averaging profits from the decreasing standard error of the mean, which leads to more precise estimates for larger crowds (Larrick & Soll, 2006). In contrast, judgments in sequential collaboration are not aggregated algorithmically (e.g., by computing the mean or median) but can be substantially changed at each position in the sequential chain. We expect that this will result in considerable variation of judgments in longer sequential chains, because errors do not cancel out (Mayer & Heck, 2024). Thus, compared to the aggregation of independent judgments, sequential collaboration should lead to more accurate results in smaller groups (due to the implicit weighing of expertise) but to equally or less accurate results in larger groups (since errors do not cancel out).

### Simulation study

To derive predictions from the computational model for longer sequential chains, we extended our simulation above. The main interest concerns the comparison of judgment accuracy of sequential collaboration over the course of a chain against the accuracy of averaged independent judgments for equally large crowds. We expect that sequential collaboration outperforms unweighted averaging for smaller groups, whereas this pattern reverses for larger groups. We initially assumed that judgments are drawn from normal distributions with the correct answers serving as means. The same parameters were used as in the simulation above. We only extended both the number of contributors per chain and the size of crowds used for unweighted averaging to 20.

Since the materials used in previous experiments (Mayer et al., 2023; Mayer & Heck, 2024) did not yield normally distributed judgments, we also implemented a second simulation assuming skewed distributions of plausible judgments. In general, judgment distributions are often not normally distributed but can be skewed or irregular (Hueffer et al., 2013; Lorenz et al., 2011). For instance, if the task is to estimate quantities that are bounded below by zero (e.g., distances, dates, weights etc.), judgments may by overly large, resulting in a positively skewed distribution. Deviations from the normal distribution are typically accounted for by using alternative, robust measures of central tendency such as the median (Hueffer et al., 2013; Lorenz, 2021; Thomas et al., 2021) or by transforming the distribution of raw judgments before computing the mean (Lorenz et al., 2011). If the judgment distribution is skewed, averaging is often biased and does not lead to an accurate estimate of the quantity of interest. In contrast, sequential collaboration may be more successful in judgment aggregation since it assigns more weight to expert judgments, which are typically more accurate and less extreme, thus reducing the number of outliers (Mayer & Heck, 2024).

To take into account the non-normality of judgments, we performed another simulation assuming a skewed distribution. We adapted the baseline distribution of plausible judgments as well as the updated distribution of plausible judgments to be a skew-normal distribution (Ashour & Abdel-Hameed, 2010; Azzalini, 1985). The skew-normal distribution is defined as $Y \sim SN(\xi ,\omega ,\alpha )$ where $\xi$ is the location parameter, $\omega$ is the scale parameter, and $\alpha$ is the shape parameter. The location parameter $\xi$ of the skew-normal distribution is defined as $\xi = \mu - \omega \delta \sqrt{\frac{2}{\pi }}$, whereas the scale parameter $\omega$ is defined as $\omega = \frac{\sigma }{\sqrt{1-2\delta ^2/\pi }}$ where $\delta$ is $\delta = \frac{\alpha }{\sqrt{1 + \alpha ^2}}$. By assuming $\alpha = 0$, skewness is zero and the skew normal distribution becomes equivalent to the normal distribution. For $\alpha < 0$, the distribution is left-skewed, and for $\alpha > 0$, the distribution is right-skewed.

We introduce a skewness parameter $S_k$ for item $k$ to the distribution of plausible values denoting the maximum $\alpha$ entered for the distribution. $S_k$ is moderated by individuals’ expertise $E_i$ such that the judgment distribution is closer to a normal distribution for contributors with high expertise ($E_i \approx 0$). This assumption is supported empirically by judgments in the random-dots estimation task, where participants in the condition with high expertise provided judgments that were less skewed than those provided by participants in the control condition (Mayer et al., 2023). The skew-normal baseline distribution of plausible judgments (cf. Equation 1) is thus defined as

10 $$\begin{aligned} Y_{ik} \sim \text {Skew-Normal}(\xi = T_k, \omega = D_k E_i, \alpha = S_k E_i). \end{aligned}$$

In line with the modified distribution of plausible judgments, the probability of changing a presented judgment is also modified. Instead of the normal distribution, change probability depends on the cumulative distribution function of the skew-normal distribution,

11 $$\begin{aligned} P_{ik} \sim G_i + (1-G_i) 4 (\Phi (\frac{x - \xi }{\omega }) - 2T(\frac{x - \xi }{.} {\omega }, \alpha ) - 0.5)^2. \end{aligned}$$

Similarly, Equation 8, which describes the updated distribution of plausible values in sequential collaboration, is modified as

12 $$\begin{aligned} Y^*_{ik} \sim \text {Skew-Normal}(\xi = T_k + A_{ik}, \omega = D_k E_i R_k, \alpha = S_k E_i). \end{aligned}$$

Aside from these modifications, the simulation used the same parameter settings as the simulation assuming normally distributed judgments described above. We set the skewness parameter $S_k$ for an item $k$ to 1 for all 60 items $k$ simulated.

#### Results

Figure 6 display the predicted judgment accuracy in the two simulations assuming that judgments follow either a normal or a skew-normal distribution, respectively. For normally distributed judgments (A), the model predicts that unweighted averaging outperforms sequential collaboration in long sequential chains whereas the opposite pattern arose in short sequential chains. In contrast, if judgments followed a skew-normal distribution (B), the unweighted mean of independent judgments did not converge to the correct, true value. Thereby, judgments in sequential collaboration generally outperformed unweighted averaging by implicitly weighing judgments of contributors by their expertise. Moreover, for the skewed-normal distribution, the variance of judgments within each chain position remained large for both sequential collaboration and unweighted averaging. This indicates that skewness of judgments can distort aggregated estimates even for larger groups. The corresponding results for the dependent variables of change probability and change magnitude can be found in Appendix B.

### Empirical study

**Fig. 5:**
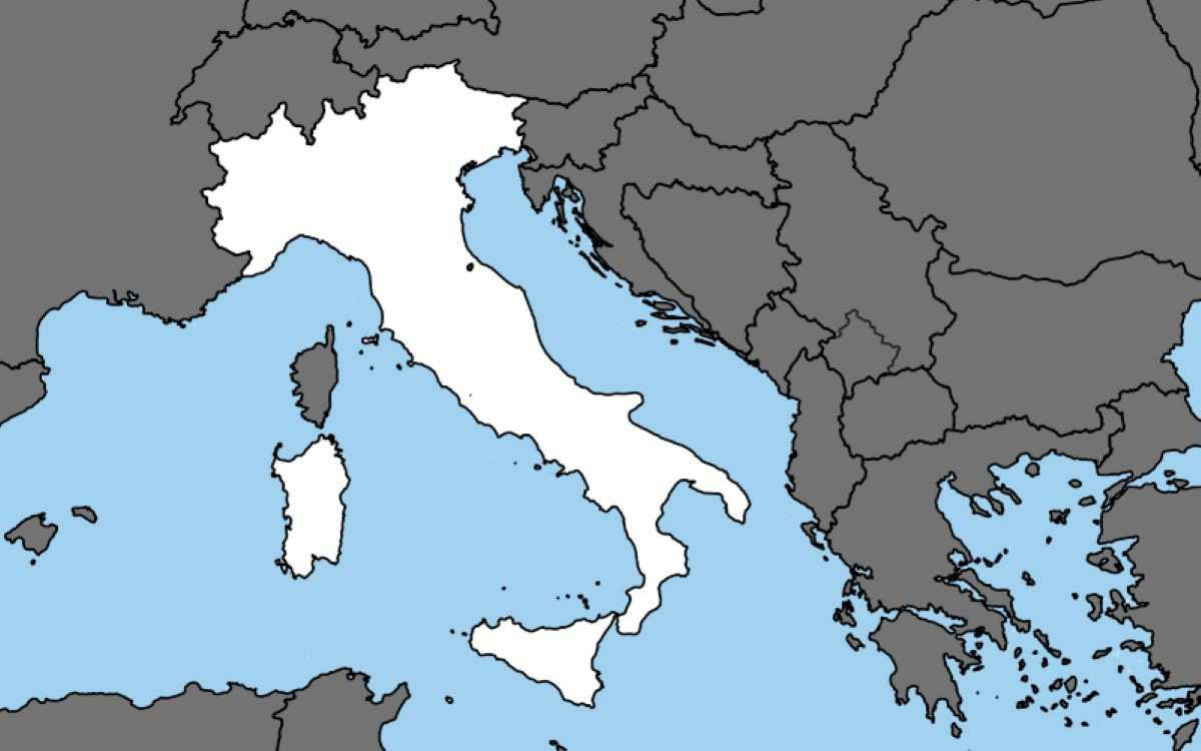
Example of the map material used in the empirical study

We tested the model predictions about the development of judgment accuracy as group size increases in an empirical study with long sequential chains of 20 contributors. We used the map materials from Experiment 3 of Mayer and Heck (2024) and added five items, resulting in 62 cities in total. The materials are known to elicit skewed judgment distributions for cities close to the borders and the sea because participants are biased towards the center of a country when positioning a city on the map. Moreover, the borders and the sea naturally restrict the possible positions of judgments (see white area in Fig. 5).

The experimental procedure of assigning participants to sequential chains was identical to that in previous studies by Mayer and Heck (2024). Participants either completed all city items independently or were presented with the judgments of previous contributors in order to form sequential chains of 20 contributors.

We collected data from 711 participants. Three participants were excluded since their chain position in a sequential chain was duplicated. Another five participants experienced technical issues during participation and were also excluded. Thus, the final sample comprises $703$ participants ($39.83$% female, $60.17$% male). Participants had a mean age of $46.34$ ($SD =17.70$) and a diverse educational background with $21.94\%$ holding a college degree, $16.52\%$ having a high school diploma, $29.20\%$ having vocational education, and $17.64\%$ having none of these educational attainments. $163$ participants were in the unweighted-averaging condition and provided independent judgments whereas $540$ participants were in the sequential collaboration condition, forming 27 sequential chains (where each chain included one participant providing independent judgments for the first position of the chain). Since only 163 participants provided independent judgments that were not used to start sequential chains, we used bootstrapping to generate 40 crowds with 20 members each as a comparison for the 27 sequential chains.

The study served both as a replication of previous results and as a test of the novel predictions of the computational model. Based on the computational model, we expected that sequential collaboration outperforms unweighted averaging especially when considering only the first few chain positions but looses its advantage in longer chains.^2^ Moreover, hypotheses for change probability and change magnitude were derived from the computational model; an analysis of these dependent variables is available in Appendix B. All hypotheses were preregistered (see https://aspredicted.org/1NJ_RWQ).

**Fig. 6:**
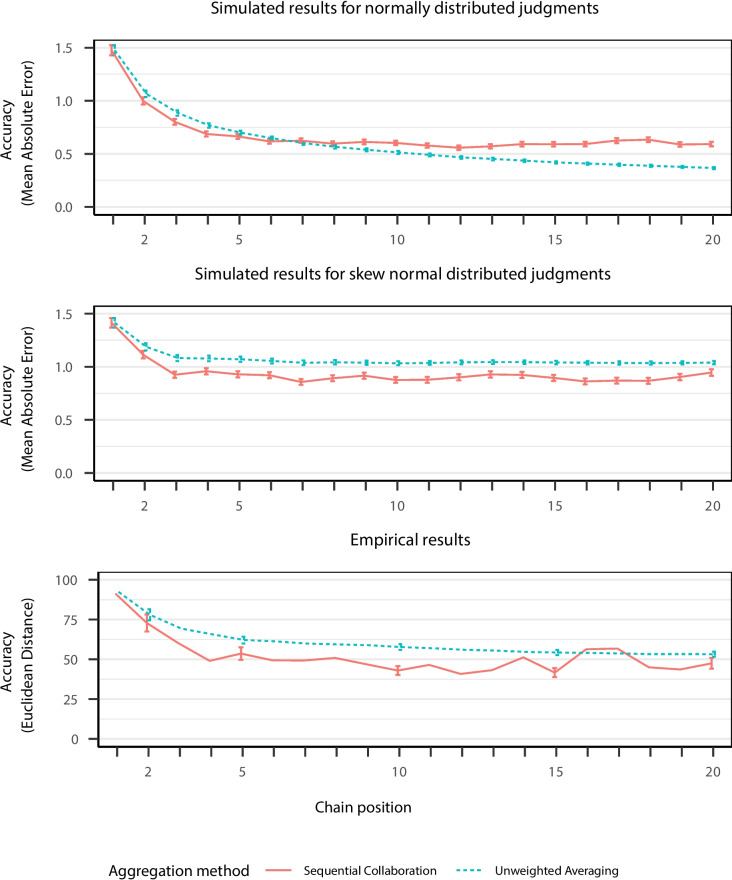
Judgment accuracy for simulated data from the computational model and empirical data for sequential chains with 20 contributors. *Note.* *Points* display empirical means with *error bars* showing the corresponding 99% between-subjects confidence intervals

#### Results and discussion

The empirical results for long sequential chains were in line with the expected patterns derived from the computational model (Fig. 6). Dependent judgments became more accurate over the course of a sequential chain. Sequential collaboration showed advantages over unweighted averaging of independent judgments especially for the first chain positions. This visual pattern is also supported by the results of a linear mixed model using chain position to predict judgment accuracy, operationalized as the Euclidean distance between location judgments and the correct position of cities. The significant negative linear trend ($\beta = -29.766$, $CI = [-43.072$, $-16.461]$, $t(536.996) = -4.385$, $p< .001$) and the significant positive quadratic trend ($\beta = 30.075$, $CI = [16.770$, $43.381]$, $t(536.996) = 4.430$, $p< .001$) indicated that errors in judgments decreased over the course of a sequential chain, especially in the beginning of sequential chains.

As expected, sequential collaboration outperformed unweighted averaging in terms of judgment accuracy for short sequential chains of six contributors ($\beta = 12.162$, $CI = [1.923$, $22.401]$, $t(62.791) = 2.328$, $p= .023$), but not for long chains of 20 contributors ($\beta = 6.255$, $CI = [-2.160$, $14.669]$, $t(58.541) = 1.457$, $p= .150$) even though the judgment distribution is skewed.

As displayed in Fig. 6, the pattern found in the empirical data resembled the pattern for simulated data assuming a skew-normal distribution of plausible and revised judgments more closely than that assuming a normal distribution. In fact, the empirical results did not show the crossed pattern in the monotonic decrease in judgment accuracy predicted for normally distributed judgments. Moreover, both the simulated and the empirical results showed that the variance in judgments in sequential collaboration remained rather large, which can be partly explained by the assumption of a skewed judgment distribution.

Overall, the empirical patterns were in line with the predictions derived from the model simulations. Sequential collaboration was best suited for smaller crowds while longer chains did not substantially increase judgment accuracy. In contrast, unweighted averaging of independent judgments is best suited for larger crowds especially if judgments are (roughly) normally distributed. Before applying either method for judgment aggregation, model predictions for the expected performance can be assessed in a Shiny Application which allows users to adjust the skewness of the underlying judgment distribution (https://marenmayer.shinyapps.io/modeling-sequential-collaboration/).

## Discussion

We proposed a computational model to formalize theoretical assumptions about the formation of dependent judgments in sequential collaboration. The model assumes that three features contribute to judgment accuracy. First, the model assumes that contributors’ expertise influences whether a presented judgment is adjusted and that high expertise reduces the variability and increases the accuracy of revised judgments. Second, in sequential collaboration, the presented judgment serves as an anchor which influences the mean of the next contributors’ distribution of plausible judgments when providing a new, revised judgment. Third, the distribution of plausible judgments is assumed to be narrower than the one for independent judgments since the presented judgment provides a frame of reference for subsequent judgments. Our simulations show that the model predicts patterns of judgments in sequential collaboration that strongly resemble empirical results. This holds both on the group level (i.e., for sequential chains of judgments) with respect to change probability, change magnitude, and judgment accuracy, as well as on the individual level with respect to the influence of contributors’ expertise on change probability and improvement of presented judgments. Besides accounting for previous results, the model also makes new predictions for long sequential chains which we tested empirically successfully.

The computational model proposed in the present paper provides a first, formalized account of sequential collaboration and reduces the ambiguity of the verbal hypotheses tested in previous work (Mayer & Heck, 2024). The two-step process assumed to underlie dependent judgments is specified in detail by modeling the decision of whether to adjust or to maintain the presented judgment as well as the result of giving a new, revised judgment. The model integrates findings and theoretical ideas from different research on numerical judgments. It assumes that expertise affects the judgment process such that individuals with higher expertise hold narrower and more accurate judgment distributions. As a consequence, sequential collaboration requires a certain degree of variation in individuals’ expertise to be successful (Mayer & Heck, 2024). If this variation is too small, judgments cannot become more accurate since all contributors equally likely opt-in or opt-out of providing a judgment (i.e., there is no implicit weighing of expertise anymore). However, if variation in expertise is large, sequential collaboration offers a high potential for eliciting accurate judgments.

Presenting a judgment of a previous participant can have advantages but it also defines boundary conditions for sequential collaboration to be successful. The model assumes that the influence of presenting a judgment of a previous contributor resembles mechanisms such as anchoring and seeding effects, which results in a smaller standard deviation of the judgment distribution. These two processes have antagonistic effects on the accuracy of judgments in sequential collaboration. On the one hand, it has been argued that anchoring poses a threat for the accuracy of dependent judgments possibly resulting in biases (Frey & Rijt, 2021; Lorenz et al., 2011; Mavrodiev & Schweitzer, 2021). On the other hand, being presented with a judgment of a different contributor serves as a frame of reference and reduces the standard deviation of revised judgments, thereby reducing the likelihood of extreme judgments. If the reduction in the standard deviation is too small, the anchoring bias results in judgments that remain inaccurate. This is due to contributors being strongly anchored by the presented judgment while they can still provide quite extreme judgments. However, possible factors influencing the amount of anchoring and the reduction in the standard deviation of dependent judgments are currently unknown and should be addressed in future research.

The model predicts that judgment accuracy improves quickly especially within the first few steps of a sequential chain. The increase in judgment accuracy is expected to be faster than for unweighted averaging of independent judgments. However, in the long run, and for larger crowds, sequential collaboration is expected to be outperformed by unweighted averaging. Sequential collaboration does not rely on averaging or any other form of mechanistic aggregation, and thus, it is more difficult to reach very high accuracy as this requires that one contributor knows the correct answer (almost) exactly. Moreover, even if an highly accurate judgment is given, it is necessary that this judgment is not further adjusted (and possibly worsened) later on by other contributors. In contrast, unweighted averaging profits from error cancellation due to the mechanistic aggregation via the mean, which leads to more stable estimates for increasing crowd sizes (Larrick & Soll, 2006).

**Fig. 7:**
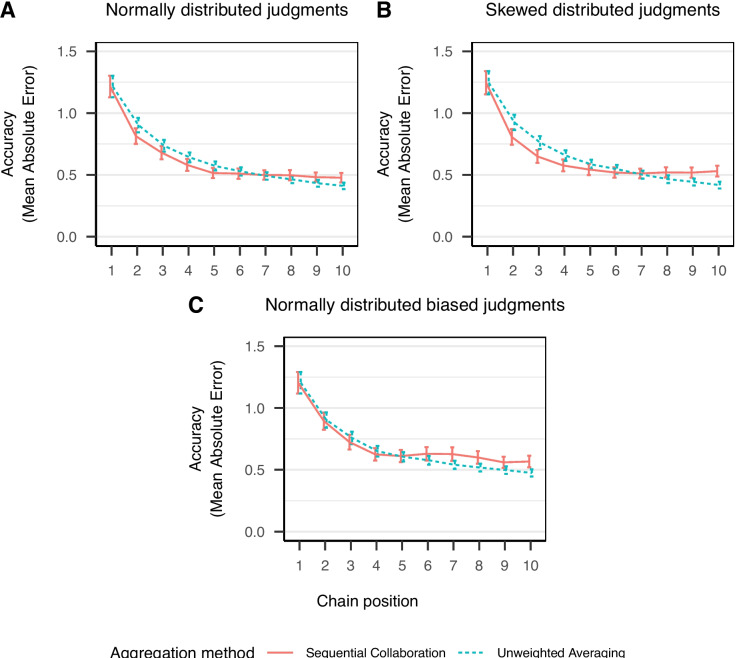
Judgment accuracy for simulated data for an unbiased normal distribution, a biased normal distribution and a skewed normal distribution of plausible judgments. *Note.* *Points* display empirical means with *error bars* showing the corresponding 99% between-subjects confidence intervals. Model parameters have been kept constant for all three simulations at $\sigma ^*_{E} = 1$, $\sigma ^*_{G} = 1$, $D_k = 3$ and $R = 0.5$ for 1000 simulated sequential chains

The proposed model assumes that the mean of the internal distribution of plausible values corresponds to the correct answer. As discussed above, this is a strong assumption that can be realistic for eliciting numerical judgments (Larrick & Soll, 2006; Steyvers et al., 2009). However, as described by Jayles et al. (2017) and Jayles et al. (2021) this may not always be the case and judgments can be biased. Above, we already studied one possible mechanism that allows to implement bias in the internal distribution of plausible judgments, namely using a skewed normal distribution. By introducing skewness, the mean of the distribution shifts away from the correct answer. The amount of skewness was dependent on individuals expertise’ such that skewness (and thus bias) reduced with increasing expertise.

An alternative approach is to assume a bias directly for the mean of the internal distribution of plausible judgments. Similar to the bias introduced via skewness of the distribution, biases affecting the mean of the distribution can be expected to be reduced by expertise since experts overall provide highly accurate judgments (Budescu & Chen, 2014; Davis-Stober et al., 2014). We extended the computational model described above with an expertise-dependent bias $B_k$ of the mean of the internal distribution of plausible judgments. The assumed amount of bias is larger for individuals with lower expertise and smaller for individuals with higher expertise:

13 $$\begin{aligned} Y_{ik} \sim \text {Normal}(\mu = T_k + B_k E_i, \sigma = D_k E_i). \end{aligned}$$

The evaluation of the plausibility of the presented judgment in sequential collaboration as well as the updated distribution of plausible values were extended accordingly.

Figure 7 displays sequential collaboration and unweighted averaging of independent judgments for A) an unbiased normal distribution, B) a biased normal distribution, and C) a skew normal distribution of plausible values. The bias was set to $B_k = 0.6$ and the skewness was set to $S_k = 0.25$ which result in the same mean $M = 0.6$ for the internal distribution of plausible values with $E_i = 1$. This reflects a bias of 20% in terms of the standard deviation. A bias induced by a skewed normal distribution results in more accurate judgments in sequential collaboration compared to unweighted averaged independent judgments over a sequential chain. In contrast, a bias of the mean of the normal distribution of plausible judgments results in less accurate judgments in sequential collaboration compared to unweighted averaged independent judgments. According to the model, the gap in performance between sequential collaboration and unweighted averaging increases as the amount of bias of the mean of the internal distribution of judgments increases. Due to the anchoring bias implemented in the model and the assumed reduction in standard deviation, large biases to the mean of the internal distribution of plausible values result in collaborative estimates that move away from the correct answer. Even though the extended computational model makes such a prediction, we do not expect this to occur in experimental settings. Instead, we assume that larger biases occur due to a combination of a bias to the mean of the underlying distribution and skewness of this distribution. Some evidence that a bias is at least partially induced by skewness has been demonstrated in our empirical study showing that a skew normal distribution best captures the response patterns of our participants.

### Limitations and future research

The computational model formalizes theoretical assumptions about sequential collaboration to derive and empirically test predictions. However, the model cannot be directly fitted to data, which limits the possibilities to directly test the validity of the model. As a remedy, a statistical model version should be developed for the computational model in the future to allow for an even more rigorous test of the theoretical assumptions about dependent judgments in sequential collaboration. Moreover, the proposed model explicitly assumes a two-step process in sequential collaboration. First, the decision whether to adjust or maintain a presented judgment is made, and second, in the former case, a new, revised judgment is provided. This is a plausible assumption and previous studies (Mayer & Heck, 2024, Experiment 2 and 3) used an experimental design mimicking this process by asking participants explicitly whether they want to adjust or maintain the presented judgment. However, these studies did not elicit judgments regarding the plausibility of the presented judgment. Future research could test whether plausibility actually matters (as assumed by the model) by asking participants to judge the plausibility of the presented judgment.

The computational model can account for central empirical findings for sequential collaboration Mayer et al. (2023) and facilitated the derivation of new, testable predictions. Here, we focused on predictions for the effects of variation in contributors’ expertise, differences in change tendency, variation in item difficulty, having shorter or longer sequential chains, and assuming normal or skewed judgment distributions. Importantly, the proposed model can be used to derive additional, new predictions. For example, the model can be used to simulate the performance of a modified version of sequential collaboration in which the possibility to opt out of giving a judgment is eliminated (Bennett et al., 2018; Bennett & Steyvers, 2022). The opt-out mechanism is an important feature of sequential collaboration (Mayer & Heck, 2024). Using the computational model as a basis, future research could disentangle the effect of opting out and that of eliciting sequential, dependent judgments. Moreover, the model can also be extended further to capture opting out for independent individual judgments (e.g., Bennett et al., 2018; Kameda et al., 2022). However, such an extension requires a separate mechanism for the decision of whether to opt out or whether to give an independent judgment. In its current version, the model is not equipped for this purpose. The opt-out mechanism for sequential collaboration relies on a comparison of the presented judgment and the internal distribution of plausible judgments. Such a mechanism cannot directly be applied for independent judgments because a presented judgment is not available. Instead, one could assume some form of absolute decision threshold of whether to give a judgment or whether to opt-out. While implementing such a mechanism goes beyond the scope of the present work, opting out for independent individual judgments is a valuable model extension that should be considered in future research.

Beyond sequential collaboration, the proposed model may also be useful for theory building in related fields. The instructions and the task in sequential collaboration share various features with paradigms commonly used to study anchoring (Tversky & Kahneman, 1974), advice taking (Bonaccio & Dalal, 2006), hindsight bias (Pohl, 2007), and seeding effects (Brown & Siegler, 1996). Even though participants taking part in experiments on such phenomena typically do not have the opportunity to refrain from providing a (revised) judgment (i.e., to opt-out), they are first presented with a numerical value before making an own judgment. The presented information can be an irrelevant value for the task at hand (as in anchoring), a closely related judgment of another participant (advice taking), the correct answer to a related task (seeding effects), or a previous judgment by oneself (hindsight bias). The proposed model of sequential collaboration provides a general formal framework of how presented information is incorporated into subsequent judgments. Note that we already considered parameters that influence the accuracy of dependent judgments such as individuals’ expertise or the presented judgments’ influence on the distribution of revised judgments. Thus, the computational model of sequential collaboration may not only shed light on the mechanisms underlying sequential collaboration but may also provide a basis for developing computational models for other forms of numerical judgments.

## Conclusion

Distilling a formal computational model from a verbal theory is considered to be an increasingly important part of theory building (Smaldino, 2017). We proposed a computational model of sequential collaboration that specifies how individuals’ expertise and general tendency to modify presented judgments contribute to maintaining and adjusting numerical judgments in sequential collaboration. The model cannot only account for previous findings but also allows researchers to derive and test new predictions. Thereby, the model provides an important contribution to theory building for sequential collaboration and for dependent numerical judgments in general.

## Figures and Tables

**Fig. 8:**
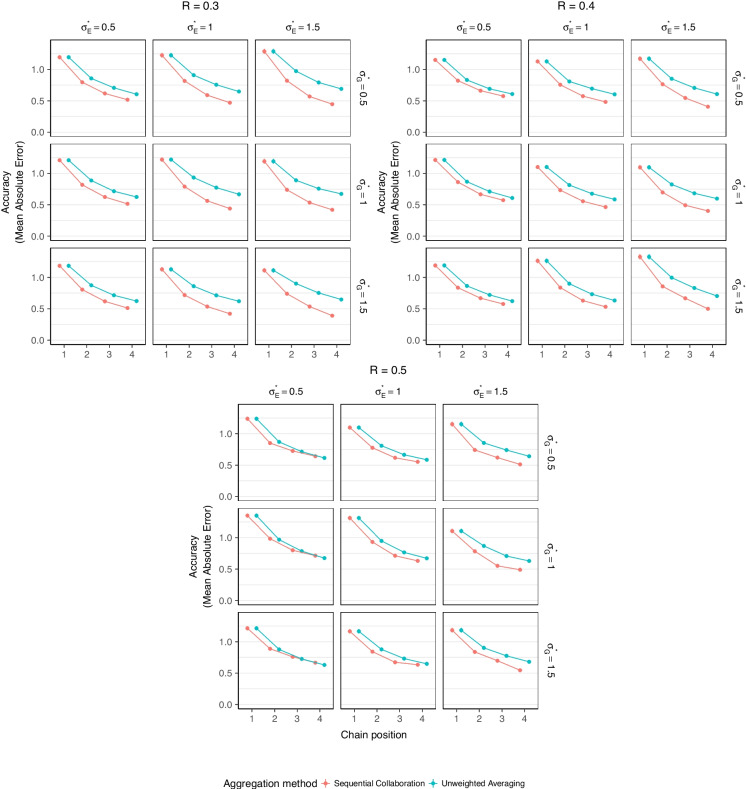
Judgment accuracy for a simulation varying $\sigma ^*_E$, $\sigma ^*_G$, and *R*. *Note.* *Points* display empirical means with *error bars* showing the (mostly not visible) corresponding 99% between-subjects confidence intervals

**Fig. 9:**
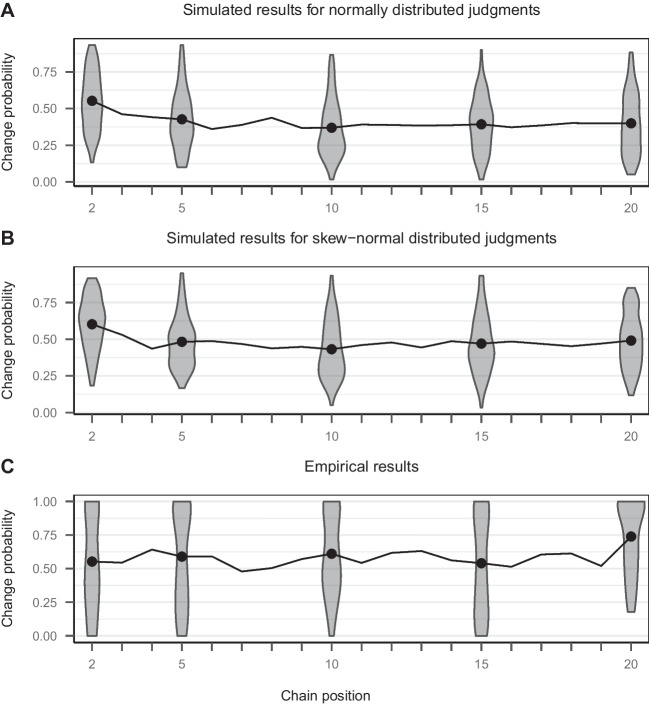
Change probability for simulated data from the computational model and empirical data for sequential chains with 20 contributors. *Note.* *Points* display empirical means with *error bars* showing the corresponding 99% between-subjects confidence intervals. *Violin plots *indicate the distribution of the dependent variable aggregated across items within each contributor

**Fig. 10:**
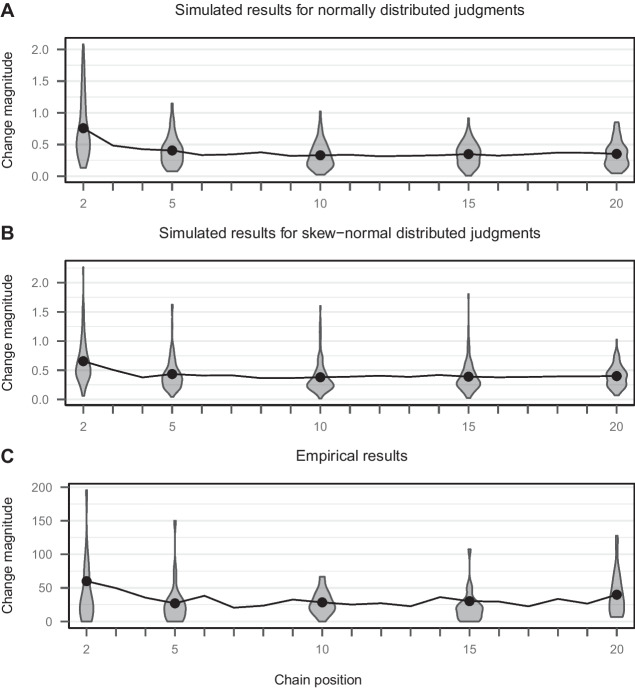
Change magnitude for simulated data from the computational model and empirical data for sequential chains with 20 contributors. *Note.* *Points* display empirical means with *error bars* showing the corresponding 99% between-subjects confidence intervals. *Violin plots* indicate the distribution of the dependent variable aggregated across items within each contributor

## Data Availability

Data and materials are available at https://osf.io/8h756/.

## Appendix B: Change probability and change magnitude in long sequential chains

The simulated results for both normally and skew-normal distributed judgments show that change probability decreases over the first five chain positions, more so for normally distributed judgments (Fig. 9 A and B). However, change probability does not converge to zero and remains on an overall higher level for skew-normally distributed judgments. Thus, we expected that the change probability for empirical data also decreases over the course of a sequential chain, however, converging to a change rate above zero. We computed a generalized linear mixed model with whether a presented judgment was adjusted (coded as 1) or not (coded as 0) as dependent variable and a linear contrast for chain position as independent variable and random intercepts for participants and items. However, the model did not show a significant effect of chain position on change probability ($\beta = 0.758$, $CI = [-0.596$, $2.113]$, $z = 1.097$, $p = .273$). This is also displayed in Fig. 9C showing that change probability fluctuating around 0.6 with no trend in any direction.

For change magnitude simulated results for both normally (Fig. 10A) and skew-normal distributed judgments (Fig. 10B) again indicate a similar pattern. Similar to change probability, change magnitude, change magnitude is expected to decrease for the first chain positions but then remains constant and does not converge to zero. This pattern was also observed empirically with Fig. 10C showing a decrease in change magnitude for the first five chain positions before chain magnitude remains mostly constant. With the increase in change probability for later chain positions, also change magnitude somewhat increases for later chain positions. Supporting the overall pattern, a linear mixed model with change magnitude as dependent and chain position as independent variable revealed a significant negative linear contrast for chain position ($\beta = -16.459$, $CI = [-32.737$, $-0.181]$, $t(510.991) = -1.982$, $p = .048$) indicating that change magnitude indeed decreases with increasing chain position.

## Footnotes

1 As adopted from cultural consensus theory (Anders et al., 2014), $E_i$ could also be referred to as an *incompetence* parameter since lower values indicate higher expertise through a narrower distribution of plausible judgments and vice versa.

2 For each sequential chain, six items were not initialized by independent judgments but rather set to the correct answer to observe adjustments to already correct answers. An analysis of these items can be found in the supplemental material at OSF (https://osf.io/8h756/).

## Code Availability

Code for the modeling, simulations, and data analyses is available at https://osf.io/8h756/.
